# Multi-Objective Optimization of Epoxy Resin Adhesive for Pavement Toughened by Self-Made Toughening Agent

**DOI:** 10.3390/polym15081946

**Published:** 2023-04-19

**Authors:** Huaiqiang Ba, Luxin Guo, Haiyang Huan, Shibo Zhang, Zhiwei Lin

**Affiliations:** 1 CRCC Yunnan Investment Co., Ltd., Kunming 650299, China; 2 Faculty of Civil Engineering and Mechanics, Kunming University of Science and Technology, Kunming 650500, China

**Keywords:** asphalt pavement, pavement materials, epoxy resin, toughening agent, mechanical properties, response surface method, gray relational analysis

## Abstract

Epoxy resin adhesive for pavement is often insufficient in flexibility and toughness. Therefore, a new type of toughening agent was prepared to overcome this shortcoming. To achieve the best toughening effect of a self-made toughening agent on an epoxy resin adhesive, its ratio to the epoxy resin needs to be optimally selected. A curing agent, a toughening agent, and an accelerator dosage were chosen as independent variables. The epoxy resin’s adhesive tensile strength, elongation at break, flexural strength, and flexural deflection were used as response values to establish a single-objective prediction model of epoxy resin mechanical property indexes. Response surface methodology (RSM) was used to determine the single-objective optimal ratio and analyze the effect of factor interaction on epoxy resin adhesive’s performance indexes. Based on principal component analysis (PCA), multi-objective optimization was performed using gray relational analysis (GRA) to construct a second-order regression prediction model between the ratio and gray relational grade (GRG) to determine the optimal ratio and to validate it. The results showed that the multi-objective optimization using response surface methodology and gray relational analysis (RSM-GRA) was more effective than the single-objective optimization model. The optimal ratio of epoxy resin adhesive was 100 parts of epoxy resin, 160.7 parts curing agent, 16.1 parts toughening agent, and 3.0 parts accelerator. The measured tensile strength was 10.75 MPa, elongation at break was 23.54%, the bending strength was 6.16 MPa, and the bending deflection was 7.15 mm. RSM-GRA has excellent accuracy for epoxy resin adhesive ratio optimization and can provide a reference for the epoxy resin system ratio optimization design of complex components.

## 1. Introduction

The steel bridge deck pavement system is an important part of the steel box girder bridge, whose waterproof adhesive layer is critical. Its failure is one of the main factors causing the problem of steel deck pavement [1]. The failure of the waterproof adhesive layer leads to the separation or slippage between the pavement layer and the bridge deck. This allows the steel structure of the steel box girder bridge to be eroded by water, thus causing serious damage to the main structure of the bridge. Using materials with excellent mechanical properties, stability, and deformability in relation to the waterproof bonding layer is a critical measure to avoid adhesive failure and to prolong the life of the steel bridge deck pavement system [2]. At present, two kinds of waterproof adhesives for steel bridge deck pavement are commonly used, namely, epoxy resin adhesive and methacrylate adhesive [3,4]. Epoxy resin adhesive has excellent mechanical properties, high bonding strength, good impermeability, strong corrosion resistance, and other advantages [5,6], and it has been extensively studied.

Zeng et al. [7] studied the components of epoxy resin binder by single factor control variable method. With the increase in the amount of toughening agent, the elongation at break increased, but the tensile strength and elastic modulus decreased. Chen et al. [8] studied the effects of different raw-material compositions on the performance of epoxy resins for road surfaces. They determined reasonable material compositions, as well as ratios, and found that the epoxy resin raw-material composition had a significant impact on the performance of epoxy resin, and its bonding performance index was proportional to the mechanical performance index. Xi et al. [9] studied three types of epoxy resins for waterproof binding and found that the tensile strength and elongation at break data for all three were quite different. Liu et al. [10] studied the basic properties of epoxy resin adhesive for steel bridge deck pavement under different environmental conditions. The tensile strength of the self-made epoxy resin adhesive was 3.7 MPa, the elongation at break could reach 180%, and it had good high- and low-temperature performance, bonding performance, and water permeability resistance. Zhou et al. [11] summarized the results of relevant research on epoxy resin adhesives. They found that the elongation at break of epoxy resin adhesives with high tensile strength is low, and their strength and deformability are often negatively correlated. How to balance the interaction of each component and how to achieve the balance of strength and deformability, two important mechanical indexes, as well as how to prepare epoxy resin with high strength, strong deformability, and good toughness as a waterproof binder, have become the foci of this research.

Response surface methodology (RSM) is an optimization method, integrating experimental design and mathematical modeling proposed by mathematicians Box and Wilson [12], which can be used to solve multifactor and multilevel continuous response problems. Compared with orthogonal experimental and uniform designs, it has the advantage of high accuracy and can also analyze the interaction between influencing factors [13,14,15]. Epoxy resin binder has several performance indicators with different dimensions to be evaluated that are difficult to balance, which leads to the challenge of determining their optimal ratio. Gray relational analysis (GRA) is introduced to solve multi-objective response problems; it is suitable for solving those involving complex relationships among multiple objectives and factors and can optimize multi-objective responses. Compared with most scholars’ single-factor control variable method [16,17], the RSM–GRA method has the advantages of analyzing the interactions of multiple factors and obtaining the best material parameters quickly and accurately [18].

In this study, the dosage of the curing agent, self-made toughening agent, and accelerator were taken as independent variables, and the tensile strength, elongation at break, bending strength, and bending deflection of epoxy resin adhesive were taken as response values. The response surface optimization test was designed by the response surface methodology Box–Behnken design (RSM-BBD) method. RSM was used to establish the single-objective prediction model of each response. The influence of factor interactions on the response values was analyzed, and the single objective optimal ratio was determined. On this basis, the gray relational analysis (GRA) method was introduced to solve the problem of multi-objective response optimization, and the gray relational degree (gray relational grade (GRG)) prediction model was established to optimize the above indicators to obtain the optimal proportion scheme of epoxy resin binder with comprehensive properties. Our results provide a reference for the design of an epoxy resin system with complex components and may guide the further development and engineering application of self-made acrylate copolymer toughening agents.

## 2. Materials and Methods

### 2.1. Raw Materials

The epoxy resin used in the study was E-51 bisphenol A epoxy resin, whose epoxy equivalent is 184~195 g/eq, and its viscosity is 10,000~16,000 mPa∙s (25 °C), and this was produced by Baling Petrochemical Company, Sinopec., Yueyang, China. The curing agent was tung oil anhydride (TOA) with a viscosity of 5000~15,000 mPa∙s (25 °C), and the anhydride equivalent is 120, and it was produced by Shandong Jiaying Chemical Technology Co., Ltd., Qingdao, China. The accelerator was 2,4,6-tris (dimethylaminomethyl) phenol (DMP-30) with an amine value of 600~630 mg/g and viscosity of 100~300 mPa∙s (25 °C), which was produced by Changzhou Shanfeng Chemical Co., Ltd., Changzhou, China.

### 2.2. Synthetic Toughening Agent

The synthesis reaction was carried out in two steps. Step 1: polyether glycol (PTMG) was dehydrated and vacuumed at 110 °C for 2 h. Then, when the temperature was reduced to 60 °C, a certain amount of toluene diisocyanate (TDI) was added, and the molar ratio of TDI to PTMG was 2:1. The reaction was carried out at 80 °C for 2 h to obtain the NCO-terminated polyurethane prepolymer. Step 2: after the prepolymer was prepared, 1,4-butanediol diglycidyl ether (BDDGE) and a small amount of 2-ethyl-4-methylimidazole (EMI) were added successively, and the reaction temperature was kept at about 160 °C. Samples were taken every 1 h for infrared testing, and the reaction was continued until the NCO infrared absorption peak disappeared. The epoxy value of the final product was 0.23, and it was marked as the self-made epoxy resin toughening agent for pavements (SM-EPT).

### 2.3. Sample Preparation

**Figure 1:**
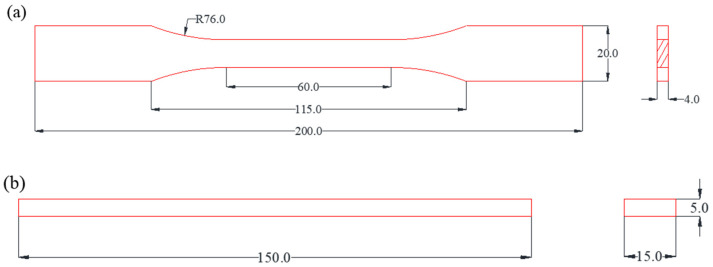
The geometric size of the specimen: (**a**) tensile test specimen, (**b**) bending test specimen.

**Figure 2:**
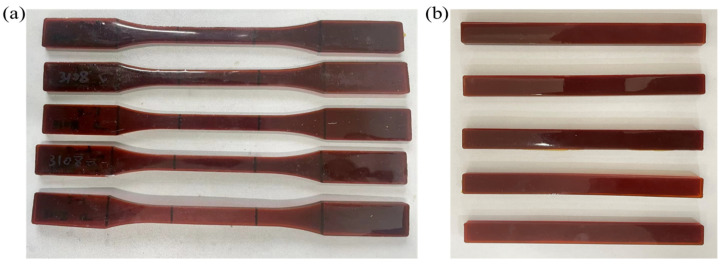
The prepared specimen: (**a**) tensile test specimen, (**b**) bending test specimen.

The epoxy resin and the toughening agent were preheated in a vacuum drying oven (DZF-1, Beijing Yongguangming Medical Instrument Co., Ltd. Beijing, China) at 80 °C and dried in a vacuum for 30 min, then mixed, and stirred at 80 °C for 5 min at 1000 R/min to obtain component A. The curing agent and the accelerator were mixed and stirred for 5 min at 80 °C and 1000 R/min to obtain component B. The components A and B were mixed and stirred for 10 min at 60 °C and 500 R/min to obtain the epoxy resin binder. Then, the binder was placed in a vacuum drying oven, defoamed at 60 °C for 20 min, and poured into a PTFE mold. The geometric size of the specimen is shown in Figure 1. It was cured at 100 °C/12 h + 120 °C/12 h in a constant-temperature drying oven (101-0ES, Beijing Yongguangming Medical Instrument Co., Ltd. Beijing, China) and then naturally cooled to room temperature for demolding and performance testing. The prepared specimen is shown in Figure 2.

### 2.4. Test Method

The mechanical properties of the specimens were tested according to the test method for properties of resin castings (GB/T 2567-2021). An electronic universal testing machine (DDL100, Changchun Research Institute of Mechanical Sciences Co., Ltd., Changchun, China) was used to continuously record the tensile and bending loads. Extensometer (YYU-25/50, Steel Yanke Testing Technology Co., Ltd., Beijing, China) was used to measure the elongation within the gauge length of the tensile test piece. The deflection at the mid-span of the bending specimen was measured by a displacement meter (JC-LVDT type, Liyang Jincheng Testing Instrument Factory, Liyang, China). The loading speed and the bending tests were 10 mm/min. Five samples were tested in each group to obtain the average value.

### 2.5. Response Surface Experimental Design

Consulting with the relevant research results [19,20,21], the dosage range of the curing agent was 150~170%, the dosage range of toughening agent was 10~20%, and the dosage of the accelerator was 2~4%. Following the RSM-BBD method, the amount of curing agent, toughening agent, and accelerator (calculated by the mass percentage of epoxy resin, represented by *A*, *B*, and *C*, respectively) were taken as the independent variables, and the tensile strength ($\sigma_{t}$), elongation at break ($\varepsilon_{t}$), bending strength ($\sigma_{f}$), and bending deflection (S) of epoxy resin adhesive were taken as the response values. A three-factor, three-level response surface test was designed. The factor code and level design are shown in Table 1. The data were analyzed by Design-Expert8.0 statistical software, and the response surface design and test results are shown in Table 2.

## 3. Results and Discussion

### 3.1. Construction of the Single-Objective Optimization Model

#### 3.1.1. Model Construction and Model Verification

Multivariate quadratic regression fitting was carried out on the test data to obtain the regression models of $\sigma_{t}$, $\varepsilon_{t}$, $\sigma_{f}$, and *S*, as shown in Equations (1)–(4).

(1) $$\begin{array}{c} \sigma_{t}=-468.105+5.302\times A+2.248\times B+21.595\times C-0.002\times A\times B-0.094\times A\\ \times C+0.170\times B\times C-0.015\times A^{2}-0.069\times B^{2}-1.159\times C^{2} \end{array}$$

(2) $$\begin{array}{c} \varepsilon_{t}=-1024.470+12.993\times A-5.158\times B+32.626\times C+0.032\times A\times B-0.037\times A\\ \times C-0.315\times B\times C-0.042\times A^{2}-0.053\times B^{2}-3.465\times C^{2} \end{array}$$

(3) $$\begin{array}{c} \sigma_{f}=-294.172+3.270\times A+0.770\times B+18.830\times C-0.003\times A\times B-0.075\times A\\ \times C-0.031\times B\times C-0.0095\times A^{2}-0.036\times B^{2}-1.004\times C^{2} \end{array}$$

(4) $$\begin{array}{c} S=-198.850+2.251\times A-0.075\times B+4.994\times C+0.005\times A\times B+0.013\times A\\ \times C-0.122\times B\times C-0.084\times A^{2}+0.0018\times B^{2}-0.824\times C^{2} \end{array}$$

where $\sigma_{t}$ is tensile strength in MPa; $\varepsilon_{t}$ is elongation at break in %; $\sigma_{f}$ is bending strength in MPa; S is bending deflection in mm; *A* is the amount of curing agent,%; *B* is the amount of toughening agent in %; and *C* is the dosage of accelerator in %;

To explore the significance of the influence between the factors (independent variables) of the response surface regression model and the response values, the regression model was subjected to analysis of variance, as shown in Table 3. The model was evaluated using the *F* test. The larger the *F* value is, the smaller the *p* value is, which means that the probability of the invalid hypothesis of the model is also smaller, and the model is more significant [22]. The *F* values of the regression models $\sigma_{t}$, $\varepsilon_{t}$, $\sigma_{f}$, and S were 66.50, 49.57, 46.32, and 40.49, respectively, and the corresponding *p* values were less than 0.0001, indicating that the statistical significance of the four models was very high. The *p* values, corresponding to the *F* values of the four regression models, were greater than 0.05, indicating that the lack of fit caused by error was not significant. The four models could well describe the relationship between response and factors.

Table 3 shows the relationship between three independent variables (factors) and materials’ response values. It can be seen that the order of influence of the three factors *A* (curing agent dosage), *B* (toughening agent dosage), and *C* (accelerator dosage) on the tensile strength model was *A* > *B* > *C*, the order of influence of the three factors on the bending strength model was *A* > *C* > *B*, and factor *A* was the most significant in both models. The results show that the amount of curing agent was the main factor affecting the strength of epoxy resin adhesive. *A*, *B*, and *C* influenced the elongation at break model in the order of *B* > *C* > *A* and influenced the bending deflection model in the order of *B* > *A* > *C*. Factor *B* was the most significant factor in both models, which indicates that the content of toughening agent was the main factor affecting the deformation ability of epoxy resin adhesive. In the two models of tensile strength and flexural strength, *AC* was significant (*p* < 0.05), while *AB* and *BC* were not (*p* > 0.05), indicating that the interaction of curing agent dosage *A* and accelerator dosage *C* had significant effects on tensile strength and flexural strength. The order of factor interaction significance in the elongation at break model was *AB* > *BC* > *AC*, and the order of factor interaction significance in the bending deflection model was *BC* > *AB* > *AC*.

**Figure 3:**
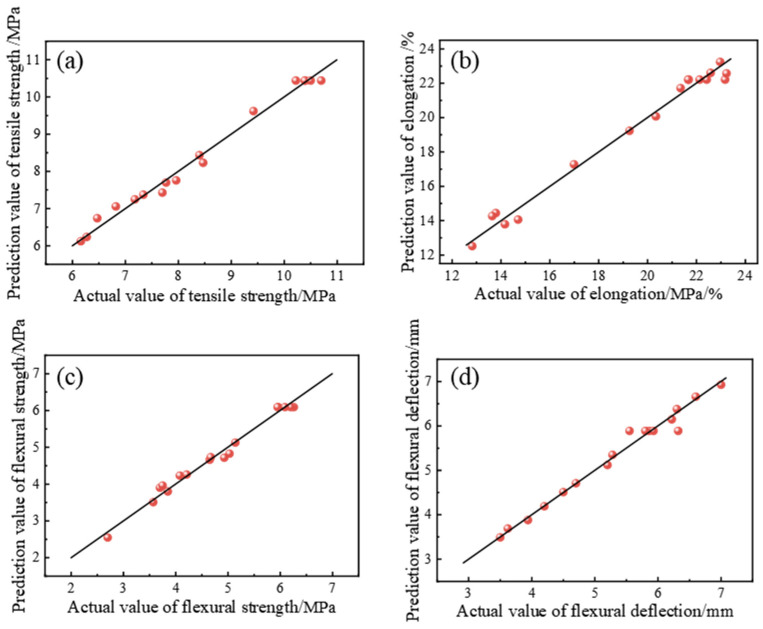
Comparison of actual and prediction values: (**a**) model $\sigma_{t}$, (**b**) model $\varepsilon_{t}$, (**c**) model $\sigma_{f}$, and (**d**) model S.

Table 4 shows the statistical analysis of the fitting accuracy of the four regression models $\sigma_{t}$, $\varepsilon_{t}$, $\sigma_{f}$, and S. The R^2^ of the four models is close to 1, indicating that the correlation between the predicted value and the actual value of the four regression models was good. The difference between the calibration coefficient of determination (R^2^_Adj_) and the prediction coefficient of determination (R^2^_Pred_) of the four models was less than 0.2, and the coefficient of variation (CV) for all of them was less than 10%. The signal-to-noise ratio was far greater than 4, which further shows that the fitting error of the four regression models $\sigma_{t}$, $\varepsilon_{t}$, $\sigma_{f}$, and S was small, and the model fitting effect was good [23,24]. Figure 3 shows the comparison results of actual values and predicted values of models $\sigma_{t}$, $\varepsilon_{t}$, $\sigma_{f}$, and S. The predicted values of the four regression models are close to the actual values, and the average deviations between the actual values and the predicted values are 1.68%, 2.23%, 2.57%, and 1.60%, respectively, indicating that the reliability of the model fitting is high. The above analysis shows that the four regression models can accurately describe the functional relationship between the response values and the factors and analyze and predict the test results.

#### 3.1.2. Analysis of Three-Dimensional Response Surface Interaction Effect

According to the test results and the variance analysis results in Table 3, the tensile strength $\sigma_{t}$, elongation at break $\varepsilon_{t}$, bending strength $\sigma_{f}$, and bending deflection S of the epoxy resin adhesive were all affected by the interaction between the factors.

**Figure 4:**
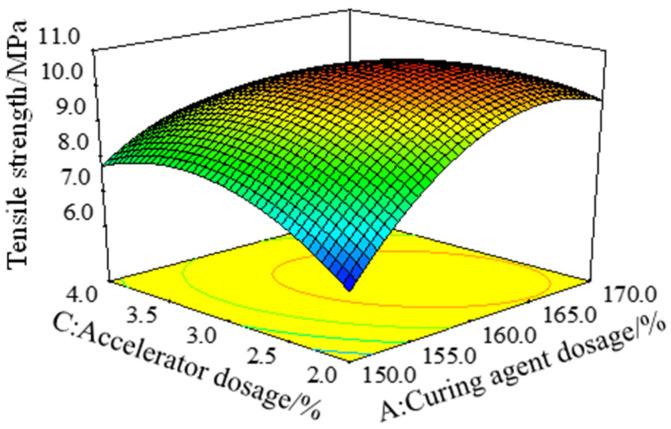
Response surface of the tensile strength model ($\sigma_{t}$) under the interaction of factors *A* and *C*.

In the regression model of tensile strength $\sigma_{t}$, the *F* value of the interaction term *AC* was the largest, and the *p* value was the smallest, which indicates that the interaction of curing agent dosage *A* and accelerator dosage *C* had the most significant effect on the tensile strength of epoxy resin adhesive. Figure 4 is a three-dimensional response surface showing the effect of the interaction between the curing agent dosage *A* and the accelerator dosage *C* on the tensile strength ($\sigma_{t}$), with the toughening agent dosage *B* of 15%. When the amount of accelerator was constant, the tensile strength increased first and then decreased with the increase in the amount of curing agent. The main reason is that when the amount of curing agent is too small, the proportion of epoxy is too high, and a large number of ether bonds are formed in the reaction process, resulting in many irreversible cross-linking points. Thus, the transesterification reaction does not occur easily in the curing system. When the cross-linking density and uniformity of the curing system were reduced, strength decreased [25,26]. When the amount of curing agent was constant, the tensile strength of epoxy resin binder increased first and then decreased with the increase in the amount of accelerator, which is consistent with the research results of Liang Ming et al. [19]. The reason is that a proper amount of DMP-30 accelerator can catalyze the anhydride group, promote the curing and cross-linking of epoxy resin, and relatively improve its mechanical properties. However, the excessive amount of accelerator will make the curing system release too much heat in unit time, resulting in phase separation of the curing system, reducing the cross-linking density, and, thus, reducing the mechanical properties of the resin [27,28,29]. In summary, simultaneously increasing the amount of curing agent and accelerator within a certain range can effectively improve the tensile strength of epoxy resin binder.

**Figure 5:**
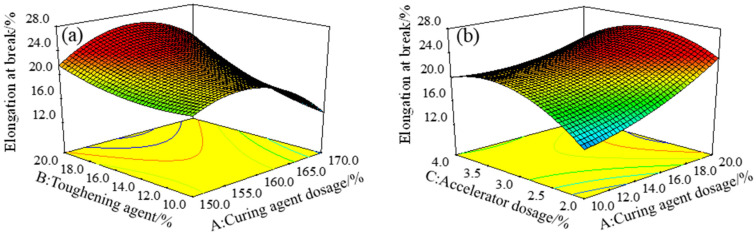
Response surface of elongation at break ($\varepsilon_{t}$) under the (**a**) interaction of factors *A* and *B*, as well as the (**b**) interaction of factors *A* and *C*.

Figure 5a is a three-dimensional response surface, showing the effect of the interaction between the curing agent dosage *A* and the toughening agent dosage *B* on the elongation at break ($\varepsilon_{t}$), with the accelerator dosage *C* of 3%. Figure 5b is a three-dimensional response surface of the effect of the interaction between the toughening agent content *B* and the accelerator content *C* on the elongation at break ($\varepsilon_{t}$), with a curing agent content of 160%. When the dosage of toughening agent *B* was constant, the elongation at break increased first and then decreased with the increase in the dosage of curing agent *A* or the dosage of accelerator *C*. When the dosage of curing agent *A* or accelerator *C* was constant, the elongation at break increased with the increase in the dosage of the toughening agent. The variance analysis of the model $\varepsilon_{t}$ results from Table 3 obtained the *p* values of 0.0036 and 0.0037 for *AB* and *BC*, respectively, indicating that the interaction of toughening agent B and curing agent *A*, as well as the interaction between toughening agent *B* and accelerator *C*, had significant effects on the elongation at break. The interaction between toughening agent dosage *B* and curing agent dosage *A* had the most significant effect on the elongation at break. *A* and *B*, as well as *B* and *C* interactions, had significant effects on elongation at break, which may be due to the decrease in cross-link density of the curing system when the toughening agent is used, but the increase in cross-link density can be caused by the addition of proper amounts of curing agent and accelerator. The interactions between the amount of toughening agent and the amount of curing agent, and between the amount of toughening agent and the accelerator, significantly affected the elongation at break [30,31]. Thus, in summary, to improve the elongation at break of epoxy resin adhesive, it is necessary to comprehensively consider the effects of the interaction of the amounts of toughening agent and curing agent, as well as the quantities of toughening agent and accelerator on the elongation at break of epoxy resin adhesive in the process of ratio optimization.

**Figure 6:**
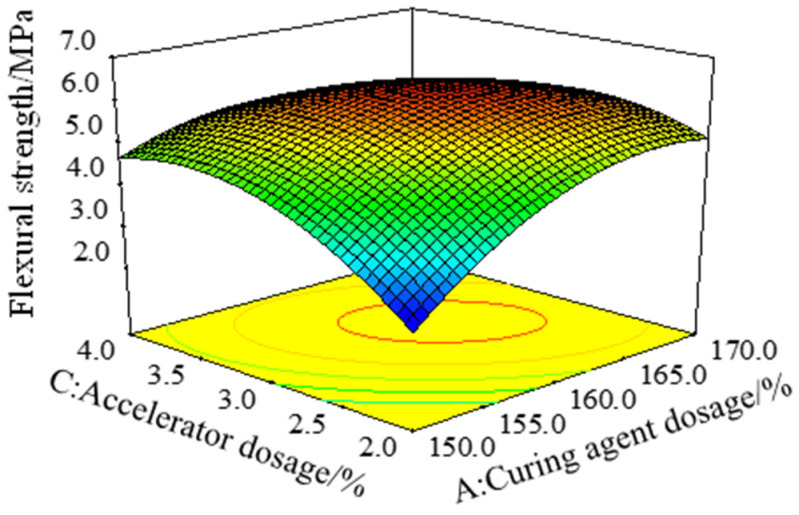
Response surface of bending strength ($\sigma_{f}$) under the interaction of factors *A* and factor *C*.

Figure 6 is a three-dimensional response surface with the effect of the interaction of the curing agent dosage *A* and the accelerator dosage *C* on the flexural strength ($\sigma_{f}$), with the toughening agent dosage *B* of 15%. It can be seen from Figure 6 that, with the increase in the amount of curing agent or accelerator, the bending strength of the epoxy resin binder increased first and then decreased. According to the variance analysis results of the $\sigma_{f}$ model in Table 3, the *AC* term was significant (*p* = 0.0002 < 0.05), and the response surface was steep when the curing agent dosage *A* and the accelerator dosage *C* changed at the same time, indicating that the interaction of the curing agent dosage *A* and the accelerator dosage *C* had a significant effect on the bending strength. The reason is that the tertiary amine produced by the decomposition of the DMP-30 accelerator can react with the anhydride group in the anhydride curing agent to produce carboxylate anion, which can catalyze the anhydride curing agent and make the system more easily cross-linked and cured. However, the excessive accelerator will quickly cross-link and cure the system, resulting in the unreacted chain segment that cannot continue participating in the reaction. Macroscopically, the interaction between the amount of DMP-30 accelerator and the amount of anhydride curing agent affects the mechanical properties of epoxy resin adhesive [19,27,28].

**Figure 7:**
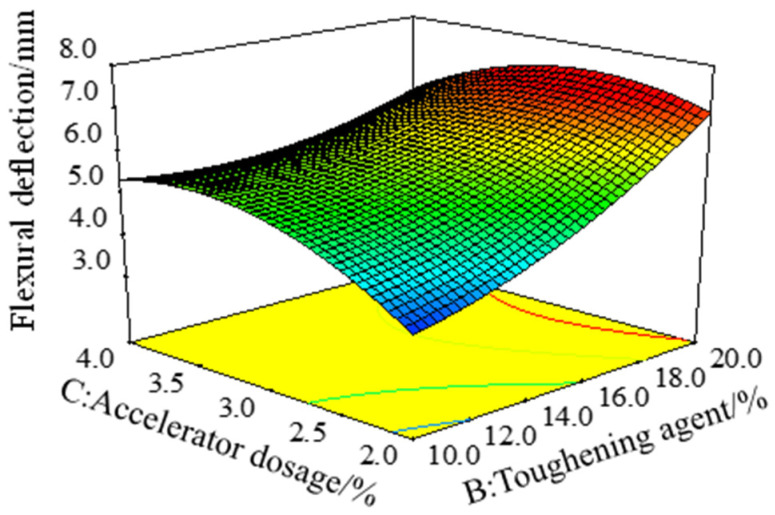
Response surface of bending deflection (S) under the interaction of factor *B* and factor *C*.

Figure 7 is a three-dimensional response surface with the effect of the interaction of the toughening agent dosage *B* and the accelerator dosage *C* on the bending deflection (S) of the epoxy resin adhesive with the curing agent dosage *A* of 160%. With the increase in the content of toughening agent, the range of change in bending deflection was greater when the content of accelerator was 2% than when it was 4%, indicating that the sensitivity of bending deflection of epoxy resin adhesive to the amount of toughening agent decreased with the increase in the amount of accelerator. According to the variance analysis results of the bending deflection S model in Table 3, the *BC* term was significant (*p* = 0.001 < 0.05), indicating that the interaction of the toughening agent B and the accelerator C had a significant effect on the bending deflection.

#### 3.1.3. Single-Objective Optimization Results

The single-objective response was optimized by Design-Expert 8.0 software. The maximum tensile strength of epoxy resin adhesive was 10.61 MPa, the corresponding amount of curing agent was 163.1%, the amount of toughening agent was 14.3%, and the amount of accelerator was 2.8%. The maximum elongation at break of the epoxy resin adhesive was 24.60%, and the corresponding amounts of curing agent, toughening agent, and accelerator were 156.9%, 18.8%, and 3.1%, respectively. The maximum bending strength of the epoxy resin adhesive was 6.17 MPa, and the amounts of curing agent, toughening agent, and accelerator were 162.5%, 14.9%, and 3.1%, respectively. The maximum bending deflection of epoxy resin adhesive was 7.42 mm, the corresponding amount of curing agent was 159.0%, the amount of toughening agent was 20.0%, and the amount of accelerator was 2.8%.

### 3.2. Construction of Multi-Objective Optimization Model

#### 3.2.1. Calculation Process of Gray Correlation Degree

Gray relational analysis (GRA) can convert multiple optimization objectives into gray relational values by reducing dimensions and then optimizing the gray relational values [32,33]. The larger the gray correlation value is, the better the corresponding response is [34]. The calculation process of the gray correlation degree is as follows:

(1) Normalization. The tensile strength ($\sigma_{t}$), elongation at break ($\varepsilon_{t}$), bending strength ($\sigma_{f}$), and bending deflection (S) of the epoxy resin adhesive were normalized to eliminate the effect of dimensions on the analysis. The bigger all four are, the better, and the normalization formula is shown in Equation (5).

(5) $$N=\frac{y-\min(y)}{\max(y)-\min(y)}$$

where N is the normalized value of each response, max(y) is the maximum value of the actual response, min(y) is the minimum value of the actual response, and y is the actual value of each group of tests.

(2) Calculation of gray correlation coefficient. The gray relational coefficient (GRC) represents the relationship between the test result and the optimal solution [35], and the calculation formulas are shown in Equations (6) and (7)

(6) $$\mathrm{GRC}=\frac{\Delta_{\min}+\xi \Delta_{\max}}{\Delta +\xi \Delta_{\max}}$$

(7) Δ=1−N

where Δ represents the deviation sequence, ξ is the judgment coefficient, ξ∈[0,1], and, in this study, ξ is 0.5.

(3) Response weight calculation. To obtain the gray correlation coefficient of each response, it is necessary to calculate the influence weight. Principal component analysis (PCA) quantitatively analyzes the weight of the contribution rate of each target to the response by reducing the dimensionality [36]. With the help of the PCA analysis module of Minitab software, the influence weights of tensile strength $\sigma_{t}$, elongation at break $\varepsilon_{t}$, bending strength $\sigma_{f}$, and bending deflection S on gray correlation degree are calculated.

(4) Calculation of gray correlation degree. Gray relational degree (GRG) is the weighted sum of gray relational coefficients. The higher the gray relational degree is, the better the corresponding response is. The calculation formula is presented in Equation (8)

(8) $$\mathrm{GRG}=\sum_{i=1}^{n}\beta_{i}\mathrm{GRC}$$

where $\sum_{i=1}^{n}\beta_{i}=1$, and $\beta_{i}$ is the weight of the ith response, calculated by PCA.

#### 3.2.2. Calculation Result and Analysis of Gray Correlation Degree

The test results are normalized by Equation (5), and GRC is calculated by Equations (6) and (7). The influence weights of tensile strength $\sigma_{t}$, elongation at break $\varepsilon_{t}$, bending strength $\sigma_{f}$, and bending deflection S on the gray correlation degree are further calculated by PCA. The results are shown in Table 5. Table 6 shows the results of the gray correlation degree calculated according to Equation (8).

#### 3.2.3. Construction and Optimization of GRG Response Model

**Figure 8:**
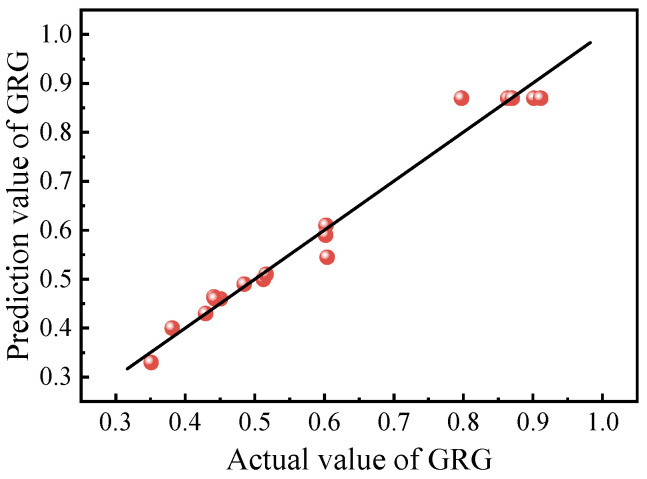
Comparison of actual and prediction values of GRG.

In order to optimize the proportion of epoxy resin binder, the mapping relationship between curing agent dosage *A*, toughening agent dosage *B*, accelerator dosage *C*, and GRG must be established. In this paper, Design-Expert8.0 software was used to establish the second-order mathematical prediction model of GRG, as shown in Equation (9). The comparison between the actual and predicted values of GRG is shown in Figure 8. It can be seen that all data are evenly distributed on a straight line and on both sides, and the average deviation between the fitting value and the actual value was 3.42%, indicating that the fitting effect of the GRG prediction model is good. According to the variance analysis results of the GRG prediction model in Table 7, the model had *p* < 0.0001, indicating that the model was highly significant. The R^2^ of the GRG prediction model was 98.39%, the difference between R^2^_Adj_ and R^2^_Pred_ was less than 0.2, and the coefficient of variation CV was less than 5%, indicating that the model had high reliability and good fitting degree and could be used for subsequent prediction and optimization.

(9) $$\begin{array}{c} \mathrm{GRG}=-68.414+0.811\times A+0.100\times B+2.204\times C+5.211\times 10^{-4}\times A\times B-5.963\times 10^{-3}\\ \times A\times C-3.427\times 10^{-3}\times B\times C-2.495\times 10^{-3}\times A^{2}-5.388\times 10^{-3}\times B^{2}-0.199\times C^{2} \end{array}$$

**Figure 9:**
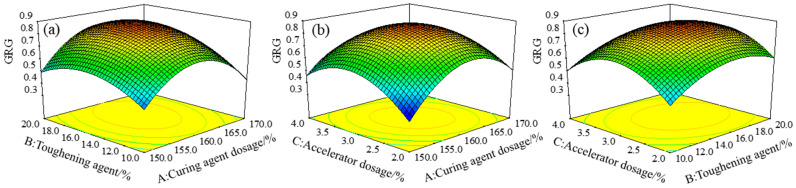
GRG three-dimensional response surface under the interaction of factors (**a**) *A* and *B*, (**b**) *A* and *C*, and (**c**) *C* and *B*.

To reflect the influence of the interaction of each factor on the GRG response value, the three-dimensional response surface of GRG under the interaction of different factors was established using the mathematical prediction model, as shown in Figure 9. The shape of the response surface of the GRG model under the interaction of different factors is a quadratic paraboloid with a downward opening, indicating a maximum value of GRG in the test range. According to the variance analysis results of the GRG response model in Table 7, the influence of the three factors on the GRG model was in the order of *B* > *A* > *C*. The interaction of curing agent dosage *A* and accelerator dosage *C* had the most significant influence on GRG. The optimized GRG response surface model was analyzed using Design-Expert 8.0 software, and the optimal gray correlation degree was 0.885, corresponding to a curing agent dosage of 160.7%, toughening agent dosage of 16.1%, and accelerator dosage of 3.0%.

### 3.3. Experimental Verification

Through the analysis of the results, the proportion of the optimal gray correlation degree (${\mathrm{GRG}}_{\max}$) was determined, and the proportion corresponding to the optimal gray correlation degree (${\mathrm{GRG}}_{\max}$) was selected for comparison with the proportion corresponding to the maximum tensile strength $\sigma_{t-\max}$, the maximum elongation at break $\varepsilon_{t-\max}$, the maximum bending strength $\sigma_{f-\max}$, and the maximum bending $S_{\max}$ deflection obtained by the single-objective prediction model.

**Figure 10:**
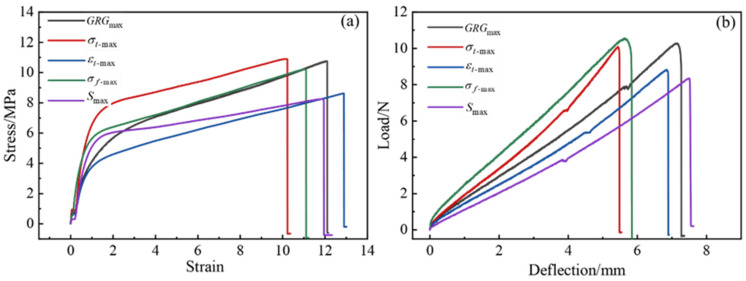
(**a**) Stress–strain curve of tensile test, (**b**) load–deflection curve of bending test.

The ratio obtained from the single-objective prediction model in Section 3.1.3 and the ratio obtained from the multi-objective optimization model in Section 3.2.3 were experimentally verified, and the experimental verification results are shown in Table 8. Figure 10 shows the stress–strain curve for the tensile test and the load-deflection curve for the bending test. The analysis revealed that the tensile strength obtained by the ratio $\sigma_{t-\max}$ was the largest, the elongation at break obtained by the ratio $\varepsilon_{t-\max}$ was the largest, the bending strength obtained by the ratio $\sigma_{f-\max}$ was the maximum, and the bending deflection obtained by the ratio $S_{\max}$ was the maximum. It can be found that the actual values of tensile strength, elongation at break, bending strength, and bending deflection deviate from the predicted values by 2.7%, 2.0%, 2.8%, and 1.1%, respectively, with actual values higher than the predicted values. This proved the feasibility of the single objective prediction model.

${\mathrm{GRG}}_{\max}$ was compared to $\sigma_{t-\max}$, elongation at break increased by 19.31%, bending strength increased by 1.81%, and bending deflection increased by 31.43%. ${\mathrm{GRG}}_{\max}$ was compared to $\varepsilon_{t-\max}$, tensile strength increased by 24.70%, bending strength increased by 16.22%, and bending deflection increased by 4.38%. ${\mathrm{GRG}}_{\max}$ was compared to $\sigma_{f-\max}$, the tensile strength was increased by 5.29%, the elongation at break was increased by 10.41%, and the bending deflection was increased by 27.00%. ${\mathrm{GRG}}_{\max}$ was compared to $S_{\max}$, the tensile strength was improved by 30.46%, the elongation at break was improved by 6.04%, and the bending strength was improved by 22.95%.

The gray relational degrees of ${\mathrm{GRG}}_{\max}$, $\sigma_{t-\max}$, $\varepsilon_{t-\max}$, $\sigma_{f-\max}$, and $S_{\max}$ were 0.830, 0.794, 0.503, 0.626, and 0.408, respectively, and the gray relational degree of ${\mathrm{GRG}}_{\max}$ was the highest. The results showed that the optimum ratio of epoxy resin binder was obtained by multi-objective optimization of response surface methodology and gray relational grade. The optimum ratio was 160.7% for the curing agent, 16.1% for the toughening agent, and 3.0% for the accelerator.

## 4. Conclusions

(1) The amount of curing agent is the main factor affecting the strength of epoxy resin adhesive, and the amount of toughening agent is the main factor affecting its deformability. The interaction of curing agent dosage and accelerator dosage has the most significant effect on the tensile strength and bending strength of epoxy resin binder. The interaction of toughening agent dosage and curing agent dosage has the most significant effect on the elongation at break of the epoxy resin binder, and the interaction of toughening agent dosage and accelerator dosage has a significant effect on bending deflection.
(2) The optimal ratio of epoxy resin binder obtained by RSM-GRA multi-objective optimization was a curing agent dosage of 160.7%, toughening agent dosage of 16.1%, and an accelerator dosage of 3.0%. An epoxy resin binder with a tensile strength of 10.75 MPa, elongation at break of 23.54%, bending strength of 6.16 MPa, and a bending deflection of 7.15 mm can be prepared with this ratio. The test results show that the RSM-GRA multi-objective optimization model is accurate, effective, and has future application significance for optimizing the epoxy resin binder ratios.
(3) Compared with the single-objective optimization model, in this study, the RSM-GRA multi-objective optimization model was used to obtain the largest gray relational grade (GRG) of the performance indicators of the epoxy resin binder, and the corresponding epoxy resin binder had the best comprehensive performance. The RSM-GRA multi-objective optimization method used in this paper can not only optimize the proportion of epoxy resin binder, but it also provide a reference for the proportion optimization of other complex epoxy resin components.

## Figures and Tables

**Table 1:** Test factors and levels.

Level	*A*	*B*	*C*
	Curing Agent/%	Toughening Agent/%	Accelerator/%
−1	150	10	2
0	160	15	3
1	170	20	4

**Table 2:** Response surface test design and test results.

NO.	Level			Test Results			
	*A*	*B*	*C*	Tensile Strength/MPa	Elongation at Break/%	Flexural Strength/MPa	Flexural Deflection/mm
1	1	1	0	7.70	22.58	5.03	6.30
2	−1	1	0	6.16	21.35	3.57	6.60
3	0	−1	1	7.77	20.34	4.93	5.28
4	0	0	0	10.70	21.68	6.26	5.86
5	−1	0	−1	6.27	14.70	2.70	4.50
6	−1	0	1	7.96	16.99	4.65	4.70
7	0	−1	−1	8.47	13.79	3.85	3.62
8	0	0	0	10.22	21.66	6.20	5.93
9	1	−1	0	8.40	14.16	4.67	3.94
10	0	0	0	10.40	23.17	5.96	6.32
11	0	0	0	10.39	22.42	6.09	5.80
12	1	0	−1	9.42	12.82	5.14	3.50
13	0	0	0	10.50	22.13	5.95	5.55
14	−1	−1	0	6.47	19.26	3.70	5.20
15	0	1	−1	7.18	22.97	3.75	7.00
16	1	0	1	7.34	13.64	4.08	4.20
17	0	1	1	6.82	23.23	4.21	6.22

**Table 3:** Results of ANOVA of response surface model.

	Model $\sigma_{t}$		Model $\varepsilon_{t}$		Model $\sigma_{f}$		Model S	
	*F*	*p*	*F*	*p*	*F*	*p*	*F*	*p*
Model	66.50	<0.0001	49.57	<0.0001	46.32	<0.0001	40.49	<0.0001
*A*	65.71	<0.0001	19.06	0.0033	52.65	0.0002	23.36	0.0019
*B*	19.28	0.0032	117.34	<0.0001	0.99	0.3526	162.85	<0.0001
*C*	3.84	0.0909	22.65	0.0021	16.81	0.0046	7.90	0.0261
*AB*	0.56	0.4804	18.44	0.0036	1.37	0.2805	4.60	0.0692
*AC*	51.89	0.0002	0.99	0.3518	51.59	0.0002	1.25	0.3009
*BC*	0.42	0.5367	18.21	0.0037	2.19	0.1825	29.70	0.0010
*A* ^2^	145.06	<0.0001	137.38	<0.0001	85.83	<0.0001	59.78	0.0001
*B* ^2^	182.64	<0.0001	13.82	0.0075	78.29	<0.0001	17.90	0.0039
*C* ^2^	82.52	<0.0001	93.06	<0.0001	96.58	<0.0001	56.98	0.0001
Lack of Fit	3.83	0.1135	1.93	0.2670	3.95	0.1086	0.47	0.9113

**Table 4:** Fit statistics for response surfaces of the model.

Model	R^2^	R^2^_Adj_	R^2^_Pred_	CV/%	Adeq. Precision
$\sigma_{t}$	0.9884	0.9736	0.8581	3.13	21.516
$\varepsilon_{t}$	0.9846	0.9647	0.8441	3.83	18.947
$\sigma_{f}$	0.9835	0.9623	0.7959	4.41	22.049
S	0.9812	0.9569	0.9398	4.20	19.992

**Table 5:** Weightiness of response.

Principal Component	Eigenvalue	Weightiness/%
$\sigma_{t}$	2.393	59.813
$\varepsilon_{t}$	1.482	37.038
$\sigma_{f}$	0.089	2.221
S	0.037	0.928
Sum	4	100

**Table 6:** Gray correlation degree calculation results.

NO.	GRC				GRG
	$\sigma_{t}$	$\varepsilon_{t}$	$\sigma_{f}$	S	
1	0.431	0.889	0.591	0.714	0.607
2	0.333	0.735	0.398	0.814	0.488
3	0.437	0.643	0.572	0.504	0.517
4	1.000	0.771	1.000	0.606	0.911
5	0.339	0.379	0.333	0.412	0.354
6	0.453	0.455	0.525	0.432	0.455
7	0.504	0.355	0.425	0.341	0.446
8	0.825	0.768	0.967	0.621	0.806
9	0.497	0.365	0.528	0.364	0.447
10	0.883	0.989	0.856	0.720	0.920
11	0.880	0.865	0.913	0.593	0.873
12	0.639	0.333	0.614	0.333	0.523
13	0.919	0.826	0.852	0.547	0.879
14	0.349	0.567	0.410	0.493	0.433
15	0.392	0.952	0.415	1.000	0.606
16	0.403	0.352	0.449	0.385	0.385
17	0.369	1.000	0.465	0.692	0.608

**Table 7:** ANOVA results for response surface model of GRG.

Source	Sum of Squares	Mean Square	*F*	*p*
Model	0.610	0.068	47.64	<0.0001
*A*	6.709 × 10^−3^	6.709 × 10^−3^	4.70	0.0668
*B*	0.027	0.027	18.98	0.0033
*C*	1.628 × 10^−4^	1.628 × 10^−4^	0.11	0.7455
*AB*	2.715 × 10^−3^	2.715 × 10^−3^	1.90	0.2103
*AC*	0.014	0.014	9.96	0.0160
*BC*	1.175 × 10^−3^	1.175 × 10^−3^	0.82	0.3945
*A* ^2^	0.26	0.26	183.57	<0.0001
*B* ^2^	0.076	0.076	53.50	0.0002
*C* ^2^	0.17	0.17	116.85	<0.0001
Error	9.991 × 10^−3^	1.428 × 10^−3^	-	-
Lack of Fit	1.820 × 10^−3^	6.068 × 10^−4^	0.30	0.8269
	R^2^ = 0.9839	R^2^_Adj_ = 0.9633	R^2^_Pred_ = 0.9327	CV/% = 6.26

**Table 8:** Response optimal ratio experimental comparison table.

NO.	*A*/%	*B*/%	*C*/%	Tensile Strength/MPa	Elongation at Break/%	Flexural Strength/MPa	Flexural Deflection/mm	GRG
${\mathrm{GRG}}_{\max}$	160.7	16.1	3.0	10.75	23.54	6.16	7.15	0.830
$\sigma_{t-\max}$	163.1	14.3	2.8	10.90	19.73	6.05	5.44	0.794
$\varepsilon_{t-\max}$	156.9	18.8	3.1	8.62	25.10	5.30	6.85	0.503
$\sigma_{f-\max}$	162.5	14.9	3.1	10.21	21.32	6.34	5.63	0.626
$S_{\max}$	159.0	20.0	2.8	8.24	22.20	5.01	7.50	0.408

## Data Availability

Not applicable.

## References

[1] Zhang Z.Q., Tao J., Zhang S.T. (2011). Experiment and evaluation on performance of epoxy asphalt waterproof cohesive layer on bridge deck pavement. J. Chang. Univ. (Nat. Sci. Ed.).

[2] Xu Y., Lv X., Ma C., Liang F., Qi J., Chou Z., Xu S. (2021). Shear Fatigue Performance of Epoxy Resin Waterproof Adhesive Layer on Steel Bridge Deck Pavement. Front. Mater..

[3] Kumar P., Patnaik A., Chaudhary S. (2017). A review on application of structural adhesives in concrete and steel-concrete composite and factors influencing the performance of composite connections. Int. J. Adhes. Adhes..

[4] Zhang M., Hao P., Men G., Liu N., Yuan G. (2021). Research on the compatibility of waterproof layer materials and asphalt mixture for steel bridge deck. Constr. Build. Mater..

[5] Xiang Q., Xiao F. (2020). Applications of epoxy materials in pavement engineering. Constr. Build. Mater..

[6] Paluvai N.R., Mohanty S., Nayak S.K. (2014). Synthesis and Modifications of Epoxy Resins and Their Composites: A Review. Polym. Plast. Technol. Eng..

[7] Zeng G.D., Wen G.X., Li J.C., Ouyang T.Z., Yuan M., Zhou Y., Li Q., Wu C.F. (2021). Development of waterproof binder for road and bridge curing flexible epoxy resin at room temperature. Highway.

[8] Chen Q., Lu Y., Wang C., Han B., Fu H. (2022). Effect of raw material composition on the working performance of waterborne epoxy resin for road. Int. J. Pavement Eng..

[9] Xi L., Huang W.R. (2016). Study on the application of epoxy resin in waterproofing layer of bridge deck pavement. J. China Foreign Highw..

[10] Liu P., Liu Y.G., Hao Z.H., Sheng X.Y., Li L. (2019). Effect of environmental conditions on bonding performance of self-made epoxy resin adhesive. Appl. Chem. Ind..

[11] Zhou L., Zhang D., Li X., Gao Z., Chen Q., Wang C. (2022). Overview: Application of Resin Waterproof Adhesive Materials in Bridge Deck Pavement in China. Adv. Civ. Eng..

[12] Box G.E., Wilson K.B. (1992). On the experimental attainment of optimum conditions. Breakthroughs in Statistics: Methodology and Distribution.

[13] Li L., Zhang Q., He Q., Hu X.B. (2015). Application of response surface methodology in experiment design and optimization. Res. Explor. Lab..

[14] Cheng J.L., Zheng M., Lou J.Q. (2012). Comparison of several common optimal experimental design methods. Res. Explor. Lab..

[15] Li Z., Lu D., Gao X. (2021). Optimization of mixture proportions by statistical experimental design using response surface method—A review. J. Build. Eng..

[16] Chen X.Y., Huang W.D., Zhang W.J., Lai Z.P., Lian G.F. (2020). Multiple targets technology optimization based grey relative analysis of 18Ni300 die steel formed by selective laser melting. Chin. J. Lasers.

[17] Singh O.P., Kumar G., Kumar M. (2019). Role of Taguchi and grey relational method in optimization of machining parameters of different materials: A review. Acta Electron. Malays. (AEM).

[18] Deshmukh S.S., Jadhav V.S., Shrivastava R. Review on Single and Multi-objective Optimization Process Parameters of EDM Using Taguchi Method and Grey Relational Analysis. Proceedings of the 9th International Conference of Materials Processing and Characterization (ICMPC).

[19] Liang M., Su L.P., Qiu Z.M., Xin X., Yao Z.Y., Ma C.Y., Ding X.M. (2021). Effects of DMP-30 on curing kinetics and mechanical properties of epoxy resin/anhydride system. J. China Univ. Pet. (Ed. Nat. Sci.).

[20] Fu P., Tan X., Xiao L.H., Nie X.A., Huang J.R., Zhang L. (2021). Study on high-performance tung oil-based anhydride epoxy curing agent. Resin.

[21] Zou S.L., Jiang S.F., Shen R.D. (2016). Properties of phenolic/epoxy resin curing system modified with acrylic block copolymer. Adhesion.

[22] Wang J.W., Wang W. (2019). Response surface based multi-objective optimization of basalt fiber reinforced foamed concrete. Mater. Rep..

[23] He D.P., Pan Z.Q., Wang H.G., Du W.X. (2022). Optimizing the content of activated crumb rubber and SBR composite modified asphalt by response surface methodology-grey relation analysis. N. Chem. Mater..

[24] Van Den Bergh D., Van Doorn J., Marsman M., Draws T., van Kesteren E.-J., Derks K., Dablander F., Gronau Q.F., Kucharský Š., Gupta A.R.K.N. (2020). A Tutorial on Conducting and Interpreting a Bayesian ANOVA in JASP. Annee Psychol..

[25] Zhou D.W., Zhai J.X., Li S., Quan X.J., Huo F., Li G. (2017). Study on curing behavior and solubility of epoxy/anhydride/DMPA system. Chem. Res. Appl..

[26] Smallenburg F., Leibler L., Sciortino F. (2013). Patchy particle model for vitrimers. Phys. Rev. Lett..

[27] Han Y., Wang Z., Zhao S., Wang J. (2019). AC impedance function of electrochemical working station as novel curing degree monitor method: A model curing system of epoxy/anhydride/DMP-30. Measurement.

[28] Huang C., Ge Z., Zhao B.B., Wang Z., Luo Y.J. (2017). Effects of DMP-30 on curing behavior of epoxy resin/maleicanhydride systems. J. Chem. Eng. Chin. Univ..

[29] Fan M., Liu J., Li X., Jue C., Zhang J. (2013). Curing behaviors and properties of an extrinsic toughened epoxy/anhydride system and an intrinsic toughened epoxy/anhydride system. Thermochim. Acta.

[30] Liu Z.L., Li H.F., Gu J.Y., Wang D.Z., Qu C.Y., Yang H.D. (2018). Properties of epoxy resin modified with acrylic block copolymer. Polym. Mater. Sci. Eng..

[31] Heng Z., Chen Y., Zou H., Liang M. (2015). Simultaneously enhanced tensile strength and fracture toughness of epoxy resins by a poly(ethylene oxide)-block-carboxyl terminated butadiene-acrylonitrile rubber dilock copolymer. RSC Adv..

[32] Huang W., Mao X., Wu Q., Zhang J. (2023). Experimental Investigation on the Shear Characteristics of Frozen Silty Clay and Grey Relational Analysis. Sustainability.

[33] Wen P., Wang C., Chen M., Chai Z. (2023). Engineering Property Evaluation and Multiobjective Parameter Optimization of Argillaceous Gangue–Filled Subgrade Based on Grey Relational Analysis. J. Mater. Civ. Eng..

[34] Hong Q., Shi Y.Y., Lu D.N., Guo Y.M. (2019). Multi-response parameter optimization for the composite tape winding process based on grey relational analysis and response surface methodology. Acta Mater. Compos. Sin..

[35] Xie Z., Liu X., Liu Z., Chen P., Lai B., Zhan B., Lao J. Human Reliability Analysis of Virus Detection Equipment Based on Entropy Weighting Method and GRA. Proceedings of the 7th International Conference on Condition Monitoring of Machinery in Non-Stationary Operations (CMMNO).

[36] Jiang R.C., Ci S.K., Liu D.W., Sun H.X., Wang D.F. (2022). Ply optimization of carbon fiber reinforced plastic control arm based on grey relational analysis. Acta Mater. Compos. Sin..

